# Barriers to and facilitators of physical activity among Saudi adults: a qualitative study

**DOI:** 10.3389/fpsyg.2025.1617255

**Published:** 2025-08-13

**Authors:** Naif Albujulaya, Clare Stevinson

**Affiliations:** ^1^Department of Physical Education, College of Education, King Faisal University, Al-Ahsa, Saudi Arabia; ^2^School of Sport, Exercise and Health Sciences, Loughborough University, Loughborough, United Kingdom

**Keywords:** physical activity, barriers, facilitators, social-ecological model, Saudi Arabia, Vision2030, outdoor physical activity

## Abstract

**Introduction:**

Physical activity is widely recognized as a cornerstone of population health and well-being, yet various barriers might hinder participation in the Kingdom of Saudi Arabia despite the Saudi government efforts to promote exercise. This study aimed to explore in depth the factors influencing physical activity, with a particular focus on outdoor exercise.

**Methods:**

The study employed snowball sampling through a social media platform to recruit Saudi men and women aged 18 years or more. Exclusion criteria include individuals under 18, with disabilities or vulnerabilities, or without access to a phone or internet-connected device for interviews. Twenty-two semi-structured interviews were conducted, and data were analyzed using qualitative content analysis.

**Results:**

Findings identified societal and environmental barriers, including discouragement from others, poor facilities, and unsafe environments, negatively impacted exercise behavior, particularly among women. Despite these barriers, personal motivations, such as improving health and mental well-being, and community engagement were identified as significant motivations to physical activity.

**Conclusion:**

The evidence from this study suggest that targeted strategies and polices (at local and national level) addressing environmental and social constraints, combined with promoting community and health-based initiatives, can enhance physical activity and outdoor exercise participation in the Kingdom of Saudi Arabia.

## Introduction

Tackling physical inactivity is a global challenge that has become increasingly prevalent in today’s society due to the rise of sedentary lifestyles, technological advancements, urbanization and other factors (Mansoubi et al., 2014; World Health Organization [WHO], 2018a). According to World Health Organization (WHO) data, Americas and Eastern Mediterranean regions tend to have higher levels of physical inactivity (World Health Organization [WHO], 2018b). In the Eastern Mediterranean area, the prevalence rates of physical inactivity ranges widely, and it is noticeable that the Gulf Cooperation Council countries stand out with higher rates compared to many of the other Eastern Mediterranean countries (Sharara et al., 2018). For example, studies have reported the prevalence of physical inactivity as 67.8% in Bahrain, 58% in the United Arab Emirates and 46% in Qatar (Awad et al., 2019; Dalibalta et al., 2021; Kahan, 2015). Physical inactivity has been identified as a key cause of many non-communicable diseases in these countries (Almahmeed et al., 2012; Chaabane et al., 2020; Mabry et al., 2016; Rahim et al., 2014; Sharara et al., 2018), and tackling physical inactivity appears to represent a particularly difficult challenge in this part of the world.

In the case of Kingdom of Saudi Arabia (KSA), the recent Saudi General Authority for Statistics report (Saudi General Authority for statistics, 2021) indicates that 68.6% of Saudi adults are not physically active. To minimize the negative impacts of physical inactivity, the Saudi government has made concerted efforts to increase participation levels. For example, it has dramatically increased the proportion of spending on the sports sector in the last few years, especially following the launch of Vision2030, with spending reaching SR2.6 billion ($693 million) in 2021 (Arabnews, 2021). Vision2030 has multiple initiatives (under the Quality of Life Program2020) that aim to promote physical activity among the Saudi population (Ksa-Government, 2018).

Alongside these, there is a continuing interest among researchers in investigating the determinants of physical activity and sedentary behaviors (Sallis and Owen, 2015). Defining the determinants will likely enhance the effectiveness of programmes and interventions that aim to promote physical activity. Bauman et al. (2012) presented an adapted Socio-ecological model of the determinants of physical activity (SEM) and demonstrated that variables related to individual, interpersonal, environmental, regional or national policy and global policy influenced participation in physical activity. Thus, addressing barriers and improving facilitators at all levels will likely contribute to increases in physical activity.

The barriers to physical activity may vary between countries and regions. For example, in Europe, an analysis of the European Commission’s Eurobarometer 88.4 survey of participants from 28 European countries identified an interpersonal factor, lack of company, and individual factors, lack of motivation and disability or illness, as major barriers to being physically active (Dėdelė et al., 2022). Meanwhile, the significant facilitators of physical activity identified were interpersonal (enjoyment of exercising with others and spending time with friends), and individual (learning new skills) (Dėdelė et al., 2022).

In contrast, Benjamin and Donnelly reviewed the quantitative and qualitative literature from 2002 to 2013 in Arab countries and identified individual factors, such as lack of time and health status, environmental factors, such as hot weather and lack of facilities, and interpersonal factors, such as traditional roles for women and lack of social support, as the most significant barriers (Benjamin and Donnelly, 2013). The most important facilitators for physical activity in Arab countries were interpersonal factors (the religion of Islam and social support), and an individual factor (the desire to be slim).

In KSA, considerable research into the barriers and facilitators to physical activity among Saudi adults has been conducted. However, the greatest focus has been on specific societal groups, such as college students and clinic patients, and on certain cities, notably Riyadh (Al-Hazzaa, 2018; Chaabane et al., 2021). For college students, studies have reported individual factors namely lack of time, lack of money and academic workload, and environmental factor namely lack of facilities as the most apparent barriers (Abdel-Salam and Abdel-Khalek, 2016; Aljehani et al., 2022; Alzamil et al., 2019; ElGilany and ElMasry, 2011; Khalaf et al., 2013; Majeed, 2015; Samara et al., 2015). While, the most frequent facilitators for Saudi college students were individual factors, namely losing weight, enhancing health, and having fun, particularly among females (Al-Rafaee and Al-Hazzaa, 2001; Khalaf et al., 2013; Majeed, 2015; Samara et al., 2015). Whereas, the major barriers among patients attending primary care clinics, urban and rural primary health centers and health centers were related to individual factors which are tiredness and lack of financial resources (Al-Otaibi, 2013; AlQuaiz and Tayel, 2009; Amin et al., 2011).

There has been limited research on the barriers and facilitators to physical activity among the wider adult population of Saudi adults (Albujulaya and Stevinson, 2023; Al-Nozha et al., 2007; Alqahtani et al., 2021). The recent population-based study by Albujulaya and Stevinson (2023) identified factors related to environment as frequent barriers. As a quantitative investigation, this could not provide detail on what these involved or deeper insight into their strength and impact (Albujulaya and Stevinson, 2023). Creswell and Poth (2016) indicate that qualitative methods are the most acceptable approaches for the comprehension of the interpersonal factors that influence the health behaviors of individuals. Thus, in order to gain a better insight into the interpersonal factors and interactions with social, environmental and policy factors that directly affect physical activity, it was decided to adopt a qualitative method for this study.

Although some research has been carried out on barriers and facilitators to physical activity in KSA, the majority of them have largely concentrated on one region or one gender. In addition, the determinants specific to outdoor physical activity have not been researched in KSA. In this study, the term outdoor physical activity will be used in its broadest sense to refer to all physical activity conducted in open-air settings such as parks, streets, or outdoor sports facilities including activities like outdoor walking, running and cycling. Albujulaya and Stevinson (2023) indicated positive levels of nature relatedness among Saudi adults, suggesting that they would be receptive to outdoor exercise opportunities in natural environments. Socially, outdoor activities are considered one of the enhancers of social cohesion and connection with nature (Dickson et al., 2008). Outdoor activities have also been shown to improve mental health more than indoor activities (Gidlow et al., 2016; Pasanen et al., 2014). It is important therefore to increase understanding of the barriers to outdoor physical activity among Saudis. This study aimed to gain further insight into the individual, interpersonal, environmental and regional or national policy factors that hinder or facilitate general physical activity and more importantly outdoor physical activity among Saudi adults.

## Materials and methods

### Participants

Ethical approval was granted by Loughborough University’s Ethics Approvals (Human participants) Sub-Committee (SSEHS-2578) for a qualitative interview-based study with Saudi adults. All participants provided informed consent before taking part.

Through snowball sampling method, recruitment involved social media and was aimed at achieving a diverse sample of Saudi adult male and female adults across the different age groups from different regions. According to Benedict et al. (2019) the use of social media for recruitment has grown in recent years as it enables researchers to readily reach a larger segment of the population. Links to study information, available in both Arabic and English, were published on Twitter (now called X) through a Tweet from the author’s account on November 26, 2020. Twitter was used as a recruitment tool due to its broad reach and ability to engage diverse segments of the population. Six Saudi public figures with large social media followings on Twitter from various regions of the Kingdom were contacted and agreed to support the author by retweeting the tweet. The study information was followed by an online invitation form asking interested volunteers for their gender, age, region and contact information. Eligibility criteria required individuals to be Saudi aged 18 years at least with access to an internet connected device or telephone for interviews, while exclusion criteria includes having any disabilities or severe physical health conditions (e.g., heart disease, cancer, or chronic respiratory illness). Within the 8-weeks recruitment period, 111 individuals from different regions of the Kingdom had volunteered, of whom 93 were eligible for the study. Text messages were sent to all participants to confirm willingness to be interviewed. Forty-five individuals did not respond and 17 declined when contacted, leaving a pool of 31 volunteers. Data collection stopped after 22 interviews due to evidence of saturation. In order to assess saturation, data saturation approach was used (Fusch and Ness, 2015). In this approach, data saturation is reached when no new information (codes and concepts) is being generated and the new data mirrors what was already stated in previous data.

### Data collection

A semi-structured interview approach was chosen because it is flexible and allows the researcher to ask additional clarifying questions depending on the interviewee’s answers (Jones, 2022) or to change the order of questions (Knox and Burkard, 2009). The SEM of physical activity determinants (Bauman et al., 2012) was used to develop an interview schedule ensuring that questions encompassed individual, interpersonal, environmental and political factors (e.g., Do you think that culture can affect your performance of physical activity? How? - In your view, what kind of activities are more accessible - indoor activities or outdoor activities? Why?).

Translating the interview schedule into Arabic was necessary because all of the interviews were conducted in Arabic, and this was carried out after the final review of the interview schedule was completed. Due to the COVID-19 pandemic, all interviews were conducted remotely either by telephone or by video conferencing (18 by telephone and 4 by video conferencing). Interviews lasted between 30 and 60 min and were audio-recorded to enable transcription.

### Data analysis

Each interview was transcribed in Arabic and then translated to English as soon as possible after completion and no later than 2 days after interview completion by the first author. In addition, to maintain the integrity of the original meaning and uphold translation quality, a back-translation approach was utilized by two distinct bilingual independent translators. These steps are important elements of increasing familiarity with the content during the stages of organizing and preparing data for analysis (Creswell and Poth, 2016). The transcripts amounted to a total of 21,125 words. A qualitative content analysis process was used for processing the interview data using the three phase approach described by Elo and Kyngäs (2008). In the preparation phase, the first author started by thoroughly reviewing the interview data, in order to immerse himself in the content. After a joint discussion with the co-author, it was decided to analyze only manifest content in the data since it enhances the avoidance of subjectivity (Schreier, 2012). Sentences and complete paragraphs were defined as the units of analysis for this study, as they were deemed relevant to understanding the influencers of physical activity. Because the analysis was based on the SEM, a deductive approach was adopted to create the main categories. The organizing phase involves creating a categorization matrix based on the SEM followed by coding of data based on content. In addition, an inductive approach was used to create and define new sub-categories and concepts within the main categories to demonstrate a connection between content that generated from the data. NVivo software (Version 12.7.0, QSR International, Melbourne, Australia) was employed to facilitate the organization of data through the coding process. The reporting phase involves organizing codes based on similarity, reviewing categories, sub-categories and concepts and calculating the sum of frequency of each concept. Greatest weight was given to concepts that were cited by 25% of interviewees were analyzed. However, valuable contributions mentioned by less than 25% were also taken into consideration.

To enhance the trustworthiness of the data and the analysis, many recommended strategies were employed (Elo and Kyngäs, 2008; Graneheim and Lundman, 2004). In order to increase the credibility, a sample with diversity in gender, age and region were chosen. Second, some units of analysis such as single words were avoided due to their lack of detailed information. Third, the authors engaged in many regular discussions to review and refine the emerging sub-categories and concepts until reaching a consensus. Additionally, to increase dependability, the co-author reviewed some of the transcripts and check if the meaning of the texts matches the sub-categories and concepts.

## Results

**FIGURE 1:**
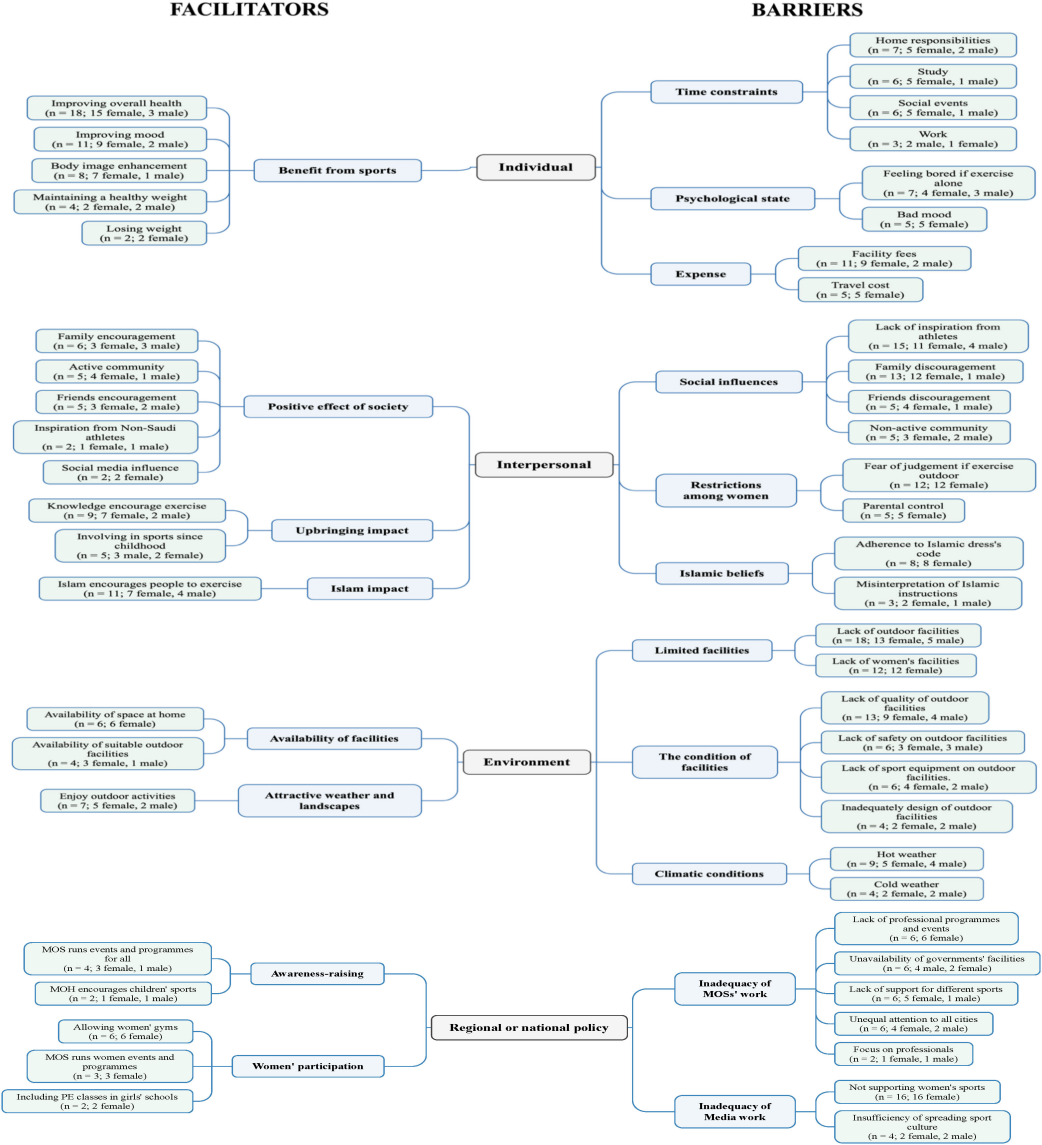
Barriers and Facilitators of Saudis’ PA. A frequency analysis is presented between the brackets to indicate the number of participants mentioning each concept.

The final sample involved twenty-two Saudi adults (six Saudi males and sixteen Saudi women with a range age of 20–65 years and a mean age of 30 ± 10.1 years). Table 1 shows each participant’s age, gender and region. The findings from the interview data were organized in the four broad categories of determinants of the SEM of physical activity: individual, interpersonal, environmental and policy. Figure 1 shows all the categories, sub-categories and concepts; in addition a frequency analysis is shown in the same figure to indicate the number of participants mentioning each concept. The sub-categories and concepts on the right side of the main categories are the barriers, while the sub-categories and concepts on the left side of the main categories are the facilitators.

**TABLE 1:** Participants’ characteristics.

Participant	Gender	Age	Region
Participant 1	Male	22	Central
Participant 2	Female	20	Central
Participant 3	Female	26	Central
Participant 4	Female	23	Central
Participant 5	Male	32	Central
Participant 6	Male	65	Central
Participant 7	Female	26	Central
Participant 8	Male	37	East
Participant 9	Female	29	East
Participant 10	Female	23	East
Participant 11	Female	24	East
Participant 12	Male	37	East
Participant 13	Female	33	South
Participant 14	Female	24	South
Participant 15	Female	28	South
Participant 16	Female	27	West
Participant 17	Female	36	West
Participant 18	Female	23	West
Participant 19	Female	25	West
Participant 20	Female	26	West
Participant 21	Male	47	North
Participant 22	Female	36	North

### Barriers

#### Individual

Individual barriers consisted of three subcategories: *time constraints, psychological state and expense*. The most frequently cited barriers in the time constraints subcategory were family/home responsibilities, study and social events. In the psychological state subcategory, the most frequently cited barrier was feeling bored if exercising alone. Lastly, facility cost was the most frequently cited barrier in the expenses subcategory.

Some participants indicated that family and home responsibilities take up a lot of their time, resulting in a lack of time for physical activity. For some men, family responsibilities were a deterrent to physical activity:

*I am married and have five children, all of whom are school age, so I have to wake up early every workday to take them to school and use my work break to pick them up. After that, I have to go shopping for household essentials and things my children need for school. In addition, I have to take my whole family to visit relatives 4 days a week. So, I spend my free time at night resting before going to bed (participant 21 - male).*

Home responsibilities for women may differ depending on their social status. For example, married women noted that taking care of children is a barrier to physical activity: “*I have four children and no one to take care of them while I am not at home*” (participant 14 - female), and “*Taking care of my children is the main barrier preventing me from exercising*” (participant 17 - female). On the other hand, single women concluded that helping their mothers with household chores such as cleaning, cooking and ironing prevents them from exercising: “*Because I am the eldest daughter, I have to help my mother with the household chores*” (participant 10 - female).

Studying is considered a barrier to physical activity for some of the participants: “*I am a master’s student, so I have a lot of work to do every day, including attending courses and writing my thesis*” (participant 16 - female). Also, other participants said that studying changes their daily schedule, making it hard for them to do physical activity: “*Studying has caused me to develop a sleep disorder because of the times of my classes, so I cannot manage to fit exercise into my day*” (participant 18 - female), and:

*I like to exercise in the morning. However, when term starts, I stop exercising because all my classes are in the late afternoon and evening, so if I wake up early in the morning, I will be tired during evening classes (participant 15 - female).*

Some women give priority to social events over physical activity, so attending family events such as weddings and everyday social events is considered a major barrier that prevents some participants from doing physical activity: “*Sometimes I cancel my plans to go to the gym because I want to meet with my cousins and other family members*” (participant 7 - female), and “*Usually family events prevent me from exercise, especially at weekends*” (participant 20 - female).

It is clear that *psychological state* is another individual obstacle that hinders physical activity. Many participants, both women and men, mentioned that lack of company to exercise with stopped them from exercising because they feel bored if they exercise alone: “*I feel bored if I do not find a group to exercise or play a sport with*” (participant 8 - male), “*I do not do any kind of sport alone because I feel bored if I do so*” (participant 1 - male), and “*I never think about exercising alone because I exercised alone before and did not complete my training due to feeling bored*” (participant 2 - female).

The expense barrier related to gym membership fees was particularly prominent in the data from the interviews with female participants: “*I would love to exercise, but gym membership fees prevent me, as they are so high*” (participant 22 - female), and “*Gyms have to reduce their membership fees, because most women cannot ask their parents or husbands for a lot of money, like me*” (participant 18 - female). Other female participants noticed that Women’s gym fees are extortionate compared to men’s:

*My husband knows the membership fees of most men’s gyms in our area, because he joined some of them in the past. When I told him that I wanted to join a women’s gym, he agreed; however, when I told him how much the membership fees were, he told me to give up on the idea (participant 7 - female).*

#### Interpersonal

Interpersonal barriers consisted of three subcategories: social influences, restrictions among women, restrictions among men and Islamic beliefs. The most frequently cited barriers in the social influences subcategory was lack of inspiration from athletes and family discouragement. Fear of judgment when exercising outdoors was reported as the most common restriction that women experience. Lastly, adherence to the Islamic women’s dress code was cited most frequently by the female participants as a significant barrier.

The participants explained how they suffer from negative influences from their social environment. Most participants, both women and men, reported that athletes’ influence on their communities is weak: “*Frankly, in KSA there are no female athletes to inspire women to do physical activity*” (participant 16 - female). Furthermore, many men feel athletes do not have a positive impact on society:

*Most professional athletes in KSA are mainly looking for money and fame, so making positive changes by promoting physical activity or spreading sports culture in society is not of interest to them (participant 6 - male).*

It was clear that families had a negative influence on exercise for female participants. Most of the female participants believed that the lack of sports culture is the main reason for their families discouraging them from taking part in physical activities: “*Because I’m skinny, my father sometimes asks me, “Why do you exercise? Do you want to disappear!*”” (participant 7 - female), and:

*My father wants to get rid of our sports equipment and always says “Enough sport!” Unfortunately, my mother agrees with my father, and she keeps saying that you can maintain your weight without sport. They both believe that sport is for obese people only (participant 16 - female).*

The majority of female participants indicated that they do not engage in physical activities, especially outdoors, due to fear of judgment from other people. As mentioned before, membership fees are so high for women’s gyms that women are forced to choose outdoor activities; however, the fear of criticism hinders them from exercising outdoors:

*I believe that my parents do not want to prevent me from doing outdoor activities, but other people’s opinions prevent them from allowing me to do so in my city. My parents said to me before “What will people say if they see you riding a bike?” (participant 19 - female).*

Clearly, this effect of society diminishes if the participants move away from their home cities or if the participants travel out of KSA: “*I used to do outdoor activities during my master’s study because I was living in a different city than my home city, and no one knew me there*” (participant 11 - female). “*I can do outdoor activities when I go to another city or out of KSA*” (participant 19 - female), and “*When I travel out of KSA, I feel more comfortable exercising outdoor*” (participant 7 - female).

Predominantly, wearing some Islamic clothes restricted female participants from doing some outdoor activities: “*I cannot practice a lot of activities in outdoor areas due to my clothes (Abayh-Hijab-Nigab)*” (participant 2 - female), and “*Wearing (Abayh-Nigab) limits me from doing any outdoor activities except slow walking*” (participant 20 - female). When participants were asked why such clothes prevented them from practicing different types of activities in outdoor areas, their answers differed. For example, some of them said that the reason for wearing these clothes was to conceal their body shapes, but most sports can expose some parts of their bodies: “*I might do any sports that do not require a lot of movement*” (participant 14 - female). Others believed that Islamic clothes were not suitable for most outdoor activities: “*It is very difficult to exercise outdoors in Islamic clothes because they might obstruct you and cause injuries*” (participant 7 - female).

#### Environment

The environmental barriers consisted of three subcategories: limited facilities, conditions of facilities and climatic conditions. A large number of participants included the lack of outdoor facilities and facilities for women in the subcategory of the availability of facilities. The most frequently cited barriers in the condition of facilities subcategory were the lack of quality of outdoor facilities, the lack of safety on outdoor facilities and the lack of sport equipment on outdoor facilities. Finally, a considerable number of participants mentioned hot weather as a barrier.

Physical environments play a substantial role in facilitating or complicating opportunities for practicing physical activities. The participants reported various barriers related to the outdoor and indoor physical environments; however, evidently, the outdoor environmental barrier constituted the largest proportion of environmental barriers. The major barrier related to outdoor environments concerned the non-availability of outdoor facilities, as mentioned by most of the participants: “*The weather attracts me to practise outdoor activities, but there are some circumstances that prevent me from doing that, and the paramount one is the non-availability of outdoor facilities*” (participant 17 - female). Similar comments included “*Outdoor facilities are largely unavailable to cover the largest proportion of the community*” (participant 5 - male), and “*Although ours is a new area, we do not have any outdoor sports facilities*” (participant 2 - female). The difficulty faced by residents in this regard was indicated by one resident:

*In our area, it was supposed to have outdoor sports facilities, such as courts and walkways, for the people of the neighborhood according to the residential plan of the area. However, we have nothing, so my neighbors and I decided to build some facilities, but the municipal authorities told us that they will be removed, as was done to other facilities that have been removed in other neighborhoods (participant 21 - male).*

Besides the lack of outdoor facilities for multi sports, some participants mentioned that even walkways were insufficient, and sometimes they had to drive around to find suitable places to walk: “*It is necessary to ensure that there are walkways in every neighborhood because we drive somewhere to practise walking*” (participant 10 - female), and “*If I want to walk in parks or any suitable outdoor facilities, I have to use my car, and usually I find parks crowded*” (participant 6 - male).

The majority of female participants highlighted that the lack of sports facilities for women hindered their participation: “*I’ve never joined a gym before because we have a few in our area and they always get overcrowded*” (participant 9 - female), “*We do not have any women’s gyms or centers in our area*” (participant 15 - female), and “*What prevents me from participating in sports is not having a place to play basketball because I used to play basketball with my friends when we were studying at the university*” (participant 3 - female).

Even though a small number of outdoor sports facilities are available, some factors negatively affect people’s desire to use them for exercise. First, the absence of quality in the existing outdoor facilities was evident, as many participants commented: “*I have to say that there is a good number of places to exercise outdoors in my city, but most of them are not in a good condition*” (participant 18 - female). Similarly: “*My physical activity is affected by the unavailability of facilities with good ground surfaces because I do not feel comfortable when I exercise on them*” (participant 1 - male), and “*The outdoor sports facilities in our area need maintenance*” (participant 3 - female). Second, some participants expressed safety concerns, such as fear of accidents and crime. Both male and female participants, when asked what prevented them from exercising outdoors, mentioned the fear of accidents as a barrier: “*Outdoor facilities must be far from the busiest streets in the city*” (participant 17 - female), “*During the lockdowns, I used to walk and run outside because there were no cars in the streets, but now I cannot*” (participant 14 - female), and:

*I almost got hit many times by cars due to lack of knowledge of the rights of bikes in streets among the people. My friend injured his back in an accident with a car while he was riding his bike (participant 8 - male).*

In contrast, female participants mentioned fear of crimes. Although KSA is recognized as a safe country, some women still feel uncomfortable exercising outdoors alone, especially at night. A female participant commented as follows: “*I become afraid if I go outside to exercise during the night*” (participant 7 - female). Third, the absence of sporting equipment is considered to be a barrier can that hinder some of the participants from exercising outdoors: “*Unfortunately, we do not have well-equipped outdoor facilities in our area*” (participant 19 - female), and “*The government should provide equipment for resistance training that suits women with religious clothing requirements (abaya and hijab)*” (participant 14 - female).

Even though the weather in KSA is extremely hot during the summer, only two of the participants indicated that they do not participate in any outdoor physical activity during the summer, with responses such as: “*I never do outdoor activities during the summer*” (participant 18 - female). Other female participants reported that the combination of hot weather and religious clothes make it difficult or uncomfortable to engage in outdoor physical activity: “*It is really difficult to exercise with a niqab in the hot weather*” (participant 7 - female), and “*In summer, the weather bothers me because the high humidity makes me struggle to breathe while I am wearing my niqab*” (participant 10 - female). Lastly, other participants highlighted that it is not possible to do outdoor activities during the daytime, but it is fine to do outdoor activities in the evening: “*I change my exercise time depending on the weather, so, in summer, I exercise after 3pm*” (participant 6 - male).

#### Regional or national policy

Regional or national policy barriers consisted of two subcategories: the *inadequacy of Ministry of Sport’s work* and the *inadequacy of the media’s work*. The most frequently cited barriers in the inadequacy of Ministry of Sport’s (MoS) work subcategory were the lack of professional programmes and events, lack of accessibility to governments’ facilities, lack of support for different sports and unequal attention to all cities. In the inadequacy of the media’s work subcategory, the most frequently cited barrier was a lack of support for women’s sport.

There is no doubt that government support for sport in general is constantly growing, but it is clear that some participants are not satisfied with the work of the MoS. Some participants acknowledged that the ministry is working to implement some programmes and events in order to raise physical activity levels among girls and to encourage them to participate, but it is clear that there is dissatisfaction with the programmes and events provided by the Ministry, as mentioned by the participants:

I know that the MoS organizes some sporting events, but, honestly, I do not participate in these events because my friends tell me that they are often overcrowded because they are not professionally organized (participant 9 - female),

The MoS should improve their events and programmes, because some people participate just for fun and to change the routine of their day. They do not continue with physical activity because they do not realize how important it is, and these events and programs fail to encourage continuity (participant 4 - female), and:

*In our city, the Ministry organizes a particular sporting event each year (let’s meet in that point and walk to the other point!) that is poorly managed, and, although many people attend this unprofessional event, I do not see how it can continue and what is the impact of this event! (participant 19 - female).*

Although the MoS owns sporting facilities in many cities, it seems that, according to some participants, the ministry does not exploit them properly: “*The Ministry should force professional sporting clubs to open their facilities for the public, especially during the off-season and to develop a community service role*” (participant 8 - male), and “*The Ministry just finished building new sporting facilities in our area, but they are not open for public use*” (participant 6 - male).

Many participants stated that the MoS’s lack of interest in supporting various sports negatively affects the practice of physical activities: “*The government should support different sports for women and not focus on only one sport (football)*” (participant 18 - female), “*I heard that the Ministry launched a women’s football league, but I do not follow its news because football is not my favorite sport*” (participant 15 - female), and “*Facilities for different sports must be provided by the Ministry because currently most of the facilities are only for football and volleyball*” (participant 1 - male).

Some participants believe that the Ministry is not making the required effort in all cities and regions, and most of the Ministry’s projects are concentrated in the largest cities: “*The ministry only concentrates on the largest cities, and they do not reach closed communities*” (participant 13 - female), “*Because the Ministry focuses only on largest cities, a change of the culture of physical activity is only happening in those cities*” (participant 9 - female), and:

*Improvements in the culture of physical activity is taking a long time to come to my city compared to other cities and regions because of a lack of interest our city. The attention of the Ministry is focused more on certain cities and regions in KSA such as Riyadh and Jeddah, so, frankly, I do not see any concrete and clear improvement (participant 16 - female).*

Interestingly, there is full consensus among female participants that the media in KSA does not support women’s sport: “The role of the media should be fulfill so a larger segment of society can be reached, especially by supporting women’s sports” (participant 4 - female), “I believe that our female athletes are not working enough to show themselves up for the public, moreover the media does not help them at all” (participant 20 - female), “I do not think that we have professional Saudi female athletes, and if we do, the media does not report on them, so how can we know them?” (participant 11 - female), and:

*Even though we know that some of the Saudi girls competed in the last Olympics, I only heard about the girl who competed against an Israeli girl through social media platforms, in addition, they made fun of her and her participation on social media (participant 13 - female).*

### Facilitators

#### Individual

As seen in Figure 1, individual facilitators consisted of one subcategory, which is the *benefits* that can be gained from participation in sport. The most frequently cited facilitator in the benefit from sports subcategory were improving overall health, improving mood and enhancing body image.

It is obvious from the data that participants believe that physical activity is essential to promote general health and prevent disease: “*When I see a man of my age using a crutch to walk, I tell myself that I do not want to end up like him. So I need to exercise and maintain my health*” (participant 6 - male), and “*My health is the primary factor that encourages me to exercise*” (participant 17 - female), and:

*Honestly, I was diagnosed with severe depression 12 years ago and exercise was one of the prescribed treatments. In addition to this, I want to improve my health and avoid medical conditions, such as diabetes and high blood pressure, which are hereditary in my family, and that I am at high risk of developing if I do not take care of myself (participant 13 - female).*

Some participants agreed that physical activity improved their overall mood and helped to reduce stress: “I exercise to put myself in a good mood” (participant 9 - female), “The only motivation for me to exercise is to get rid of my bad mood” (participant 11 - female), and “The divorce negatively affected my mental health, which has led me to view sports as an outlet for coping with psychological stress and avoiding becoming a prisoner to my negative thoughts” (participant 17 - female).

Improving physical appearance was reported as a facilitator for physical activity, and was more common among women: “*My main motivation for exercise is my body image; taking pictures and seeing improvements in my physique greatly encourages me*” (participant 16 - female), “*Staying in good shape encourages me to exercise*” (participant 14 - female), and “*I play sport because I want to be in good shape*” (participant 3 - female).

#### Interpersonal

Interpersonal facilitators consisted of three subcategories: the *positive influence of society*, the *impact of upbringing*, and the *influence of Islam*. The most frequently cited facilitator in the positive influence of society subcategory was family encouragement. While knowledge and encouragement from Islam were the most frequently cited in the last two subcategories.

Family encouragement was mentioned by many participants as a decisive facilitator in helping them to exercise and overcoming challenges that could prevent them from being active: “*Sometimes my brother encourages me to play sports, especially football. My parents also exercise, so they encourage me to exercise too in order to stay strong and healthy*” (participant 10 - female), “*Although my husband never goes to the gym (despite always paying the membership fees), he encourages me to exercise!*” (participant 14 - female), and:

*All of my family members exercise, so I would feel ashamed if I did not. Furthermore, I believe myself to be an example to my family, so if I do not exercise, I could negatively influence them. We always try to encourage each other (participant 6 - male).*

It was evident that for some participants, understanding the importance of physical activity and its beneficial effects on all aspects of life served as an impetus to stay active: “I have known the importance of exercise for a long time, so I started exercising since I was in the first year of the university” (participant 17 - female), and “I exercise because I am aware of the importance and its positive impacts on my health” (participant 15 - female).

Similarly, many participants believed that religion encourages both genders to exercise for health reasons: “*I think religion can positively affect physical activity because it encourages people to be more active*” (participant 6 - male), “*Our religion motivates people to preserve their health*” (participant 4 - female), and:

*I don’t see any negative effect of religion on physical activity at all; on the contrary, some people may use religion as a means to exercise, for example, by walking to a distant mosque (participant 1 - male).*

#### Environment

Environmental facilitators consisted of two subcategories: availability of *facilities* and the *attractive weather and landscapes*. The most frequently cited facilitator in the availability of facilities subcategory was the availability of spaces at homes. Enjoying outdoor activities in winter was cited by many participants in the attractive weather and landscapes subcategory.

Despite the lack of sports facilities, some participants noted that they sometimes did physical activity in their homes due to having access to suitable spaces:

*My sister and I do cardio and weightlifting for 40 min at home in a room that we developed ourselves by buying some sport equipment. We exercise at home because when I was 19, there were no women’s gyms in my city (participant 16 - female).*

It is worth noting that some women exercise at home not only because of the lack of sport facilities, but also because they want privacy: “*I always exercise in our yard at home because I have the freedom to do any kind of sport that I like*” (participant 19 - female).

Some participants described the positive impact of attractive weather and landscapes in encouraging them to be active: “I’m particularly drawn to nice weather, especially during winter. It encourages me to do outdoor activities” (participant 18 - female). Similar motivations were expressed: “When I visit places that have attractive weather and landscapes, I am more excited to exercise” (participant 8 - male), and “Good weather and nature (fresh air, trees, flowers, birds, wildlife, sea, sky, and scenery) have a significant impact on me, making me excited to do outdoor activities” (participant 12 - male).

#### Regional or national policy

Regional and national policy factors consisted of two subcategories: *awareness-raising* and *women’s participation*. The most frequently cited facilitator in regional or national policy facilitators subcategories were the positive impact of MoS’s programmes and events and allowing women’s gyms.

Even though some of the participants believed that the programmes and events that MoS launched lack of professionalism and effectiveness, other participants expressed an interest in participating in MoS’s programmes and events due to their positive effects on raising the awareness of the important of exercise among adults and children:

*The MoS has organized many sports programmes and events. During these programmes and events, they give gifts to participants contain flyers showing the benefits of exercise and some exercise tools such as jump ropes. I believe that these kinds of gifts promote the awareness of the important of exercise (participant 12 - male).*

Allowing women’s gyms is considered by some participants to be a radical shift in women’s sports in KSA; although, they also believe it came too late: “*Five years ago, there were no women’s gyms in our city. However, now we have a few, so girls have started to do sports there*” (participant 20 - female), and:

*About 10 years ago, there were only three women’s clubs in the Makkah region, all of which were registered as health clubs and not as gyms, because at that time gyms for women were not allowed (participant 17 - female).*

## Discussion

This study aimed to identify the factors that influence indoor and/or outdoor physical activity among Saudi adults. The findings indicate that barriers related to interpersonal and environmental factors are the most significant hindrances to participants’ physical activity. While the facilitators related to individual and interpersonal factors are the most important motivations for participants’ physical activity.

### Individual factors

Lack of time and finances obstruct an individual’s willingness to participate in physical activity. These results match those reported by previous studies, especially among college students (Abdel-Salam and Abdel-Khalek, 2016; Alzamil et al., 2019; Awadalla et al., 2014; Gawwad, 2008; Majeed, 2015). A possible explanation for these results may be that students dedicate a significant amount of time to studying and frequently lack high incomes. However, in the current study, these barriers were observed among non-students as well as students. This highlights that physical activity was not a priority among participants and that they were unable to dedicate short amounts of time to exercise. In addition, lack of company and the subscription fees for facilities were excessive so that even financially capable people refused to pay them. Therefore, the Saudi government should exploit citizens’ interest in improving their health through physical activity by gearing its programmes and events toward raising awareness among citizens that exercising, even for a short period, can improve their general health (Herbert et al., 2020; Norton et al., 2011; Pascoe et al., 2021; World Health Organization [WHO], 2018a). Furthermore, physical activity can be practiced anywhere – it is not necessary to exercise inside indoor facilities. Additionally, increasing the number of exercise groups and enabling apps that help users find virtual workout partners can facilitate physical activity.

Despite the barriers mentioned above, most participants believed that physical activity can play a substantial role in improving their mental and physical health. Similar to previous studies conducted worldwide and in Middle East countries (Benjamin and Donnelly, 2013; Chaabane et al., 2021), improving mental and physical health was identified as a facilitator for being active. This highlights the importance of publicizing the benefits of physical activity on physical and mental health among Saudis, as it has been found to be an effective means of increasing physical activity levels (Gilbert et al., 2021).

### Interpersonal factors

The negative influence of the social environment was evident, as most participants mentioned it. All participants stated that Saudi athletes fail to fulfill their social role because they do not encourage the community to be active. Despite athletes occupying significant roles in communities, since they are often role models for individuals, no research has studied the impact of athletes on communities. In addition, it is clear from the results that the government has not used athletes’ roles to promote physical activity or encourage healthy lifestyles in KSA.

For females, it is evident that family discouragement is a major obstacle that they encounter. These findings contradict those of studies that concluded that family support cannot be regarded as a major barrier and may serve as a crucial facilitator of exercise among Saudi females such as (Aljehani et al., 2022). A possible explanation for this is that the sample in the present study is from different regions while other studies sampled from only one city namely the capital, Riyadh; thus, the current study’s results may reflect variations in physical activity culture among the people of different Saudi regions. Aside from the negative influence of the social environment, the findings of this study highlight that it is difficult for females to exercise outdoors due to a fear of people talking. This finding agrees with the findings of a study conducted among Omani females (Mabry et al., 2014). It should be noted that the participants in the present study mentioned that this hindrance disappears once they leave their cities or countries. Therefore, there is a need to maximize the beneficial influence of athlete role models, raise awareness of the importance of family and the benefits of outdoor activities among Saudis in all regions and develop a plan to alter public perceptions regarding women’s participation in outdoor sports (Behnoosh et al., 2017; Chmait et al., 2020; Kilgour and Parker, 2013).

Even though many interpersonal factors were recognized by participants as barriers to physical activity, some participants highlighted that upbringing and Islam place great emphasis on being active. These findings agree with a previous study concluding that knowledge and the teachings of Islam can play a significant role in shaping Arab individuals’ attitudes toward engaging in physical activity (Benjamin and Donnelly, 2013). Therefore, the effectiveness of policies and interventions that aimed to level up physical activity rates can be strengthened by directing the efforts of social institutions such as mosques, schools and universities to spread knowledge and Islamic teachings that promote physical activity (Abdulwasi et al., 2018; Banerjee et al., 2017).

### Environmental factors

The results of this study highlight a number of environmental barriers. First, a lack of facilities, in particular, outdoor facilities and female-only facilities, was identified as the major environmental barrier among participants. The present findings seem to be consistent with the findings of two systematic reviews, which highlighted that the lack of facilities was apparent as a barrier among citizens (Al-Hazzaa, 2018; Chaabane et al., 2021). Notably though, participants in the studies within the reviews were more likely to mean indoor facilities instead of outdoor facilities such as outdoor fields, tracks, courts, cycling routes and paths, walking paths and parks. In addition, the current analysis highlights the lack of support from the relevant authorities for homeowners wishing to develop outdoor facilities in their neighborhoods and their lack of seriousness in the development of neighborhoods. With regard to the lack of female-only facilities, previous studies (Aljehani et al., 2022; Khalaf et al., 2013; Samara et al., 2015) have demonstrated similar results. Yet, these studies have indicated that participants mentioned solely the shortage of female gyms, while in the current study, participants reported the absence of both female team-sports facilities and gyms. This indicates the desire of some females to practice group outdoor sports, but the lack of privacy in the available facilities inhibits their capacity to engage in such activities. Allocating women-only days in parks combined with child-friendly amenities, may encourage greater physical activity among women and girls (Cohen et al., 2021; Pinelo Silva, 2021).

Second, notwithstanding the lack of outdoor facilities, the conditions of the available ones are unsatisfactory in several respects, which impedes individuals’ participation in outdoor activities. These findings corroborate the findings of other studies conducted worldwide (Akpinar, 2016; Kaczynski et al., 2008; Tester and Baker, 2009; Veitch et al., 2012), which have suggested that the status of outdoor facilities can impact physical activity levels. It is clear from the results of this study that facilities are deficient due to not only inappropriate infrastructure, but also inadequate locations, an absence of maintenance and a lack of sports equipment. What is surprising is that the results of the current study reveal that lack of safety in outdoor facilities is a matter of concern not only for females but also for males. In contrast to earlier findings, lack of safety in some indoor and outdoor facilities was only mentioned as a barrier among Saudi females and school students (Al-Hazzaa, 2018; Aljehani et al., 2022; Gawwad, 2008).

Third, consistent with the literature (Al-Hazzaa, 2018; Chaabane et al., 2021), the findings of the present study indicate that climate can hinder participants’ outdoor physical activity. However, the findings of the current study demonstrate that hot weather was not the main factor that prevented participants from exercising outdoors, as other studies have concluded. Instead, combined factors enhanced the influence of the weather factor, such as a lack of shaded facilities and wearing Islamic clothes (niqab) among females. Therefore, in order to overcome the impact of barriers related to environmental factors, the government should focus on developing outdoor facilities and female-only facilities while achieving high standards and providing necessary maintenance (Peyman et al., 2018).

It is clear from the results of the current study that facilitators related to environmental factors are less prominent in comparison to facilitators connected to other factors. This finding seems to be consistent with the results of a literature review that investigated barriers and facilitators influencing the physical activity of Arabic adults (Benjamin and Donnelly, 2013). Despite the scarcity of these facilitators, a conclusion can be made from the finding of the current study that developing home-based physical activity interventions can be effective among Saudi females as they offer flexibility, privacy and the ability to customize workouts according to personal preferences. In addition, the dissemination of motivational messages encouraging engagement in outdoor physical activity during periods of optimal weather conditions across the country by government agencies, or any other private agencies, may be a constructive strategy as demonstrated in different countries (Buchholz et al., 2013; Cherubini et al., 2020; De Vries et al., 2016; Kyung et al., 2024; Yan et al., 2015). This is an important area of further research for the KSA, to determine the nature of effective messaging and learning from research in other countries (Latimer et al., 2010).

### Regional or national policy factors

Participants in the present study identified some barriers related to the government’s work. Aligning with a previous study (Albujulaya et al., 2023), current results show that the shortcomings in government work were associated with two ministries, namely, the MoS and the Ministry of Media. It is worth noting that these two ministries in particular, have key roles in promoting physical activity (World Health Organization [WHO], 2018a). Therefore, these two ministries should improve the efficiency and effectiveness of their work through evidence-based actions due to the impact of Media on physical activity (Bauman and Chau, 2009). For example, these could include disseminating information related to the benefits of physical activity, expanding facilities, activating governmental facilities, creating awareness campaigns and organizing professional sports programmes and events to minimize the barriers that participants highlighted. It is somewhat surprising that despite the government’s efforts to evolve women’s sports recently, female participants expressed that governmental and non-governmental media did not support women’s sports. This suggests the need to create and implement policies intended to provide the required support to women’s sports and to people interested in women’s sports in the KSA.

The results of the present study indicate that some of the programmes and events that MoS organized benefit the population by raising the awareness of the importance of physical activity. This finding supports the idea of the positive impact of mass sporting and physical activity events in physical activity (McVinnie et al., 2023). Also, the results indicate that the provision of women’s sports facilities in the recent period contributes radically to encouraging females to practice physical activity. Therefore, enhancing physical activity events and expanding females’ sports facilities can profoundly encourage individuals to adopt a physically active lifestyle (Murphy and Bauman, 2007).

Several limitations to this study need to be acknowledged. First, only two participants were over 40, leading to a largely young adult sample and limited insight from older age groups. Second, we aimed to same number of participants from each region but encountered difficulties in recruiting from both the South and North regions. The potential self-selection bias and the underrepresentation of individuals from rural areas or cultural groups need to be acknowledged. Despite efforts to include the perspectives of males and females, the majority of the sample was actually females. The higher willingness to take part by women may reflect the subject having greater pertinence since significant obstacles to physical activity were discussed by female participants. The most likely reasons for the higher number of female participants are that women may be more affected by physical activity barriers or were more available for interviews (e.g., less likely to have a full-time work). Third, because the recruitment strategy focuses entirely on use of Twitter, non-users of Twitter were not given the opportunity to participate in this study (Benedict et al., 2019). Despite these limitations, this study is unique as one of the few qualitative studies encompassing participants from diverse regions and exploring a wide range of individual, interpersonal, environmental, and regional or national policy factors. The higher number of females in the sample might be considered a unique strength of the study as it helps address the historical underrepresentation of female voices in research on physical activity. In particular, the study represents the first comprehensive investigation to extensively explore the obstacles and facilitators of outdoor physical activity in females. Lastly, about half of the participants were generally quite active and motivated, so this provides as much insight from those who do or don’t have any interest in physical activity.

## Conclusion

It is evident that when developing new strategies and policies, greater emphasis should be placed on addressing social and environmental barriers, as they were found as more prominent and influential than other barriers. In addition, enhancing factors related to the individual and society may improve the effectiveness of future plans aimed at raising the level of physical activity. Lastly, our findings suggest that in order to enhance the physical activity levels of Saudis, it will be necessary to implement initiatives and policies that target various factors at the individual, social, environmental, and governmental levels.

## Data Availability

The datasets presented in this article are not readily available because privacy and ethical restrictions. Requests to access the datasets should be directed to Naif Albujulaya.
